# Supplementary figures and images for: Features of Mycobacterium bovis Complete Genomes Belonging to 5 Different Lineages
Source: Microorganisms. 2023 Jan 11;11(1):177. doi: 10.3390/microorganisms11010177 (PMC9865570; doi:10.3390/microorganisms11010177)

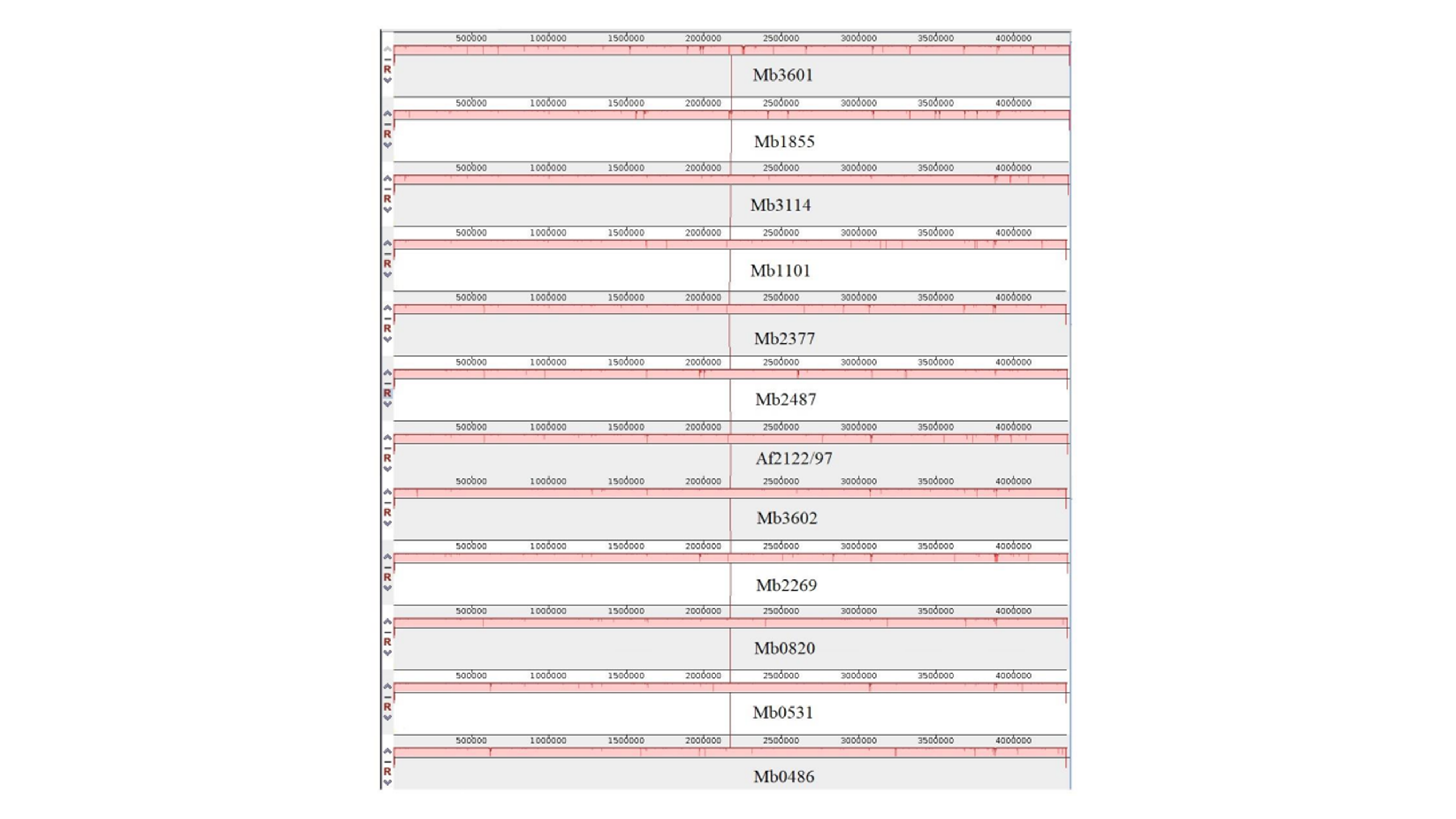

Supplement: Supplementary file 1 [file microorganisms-11-00177-s001.zip › Figure.S1.tif]
